# Autonomic Imbalance Increases the Risk for Non-alcoholic Fatty Liver Disease

**DOI:** 10.3389/fendo.2021.752944

**Published:** 2021-11-08

**Authors:** Inha Jung, Da Young Lee, Mi Yeon Lee, Hyemi Kwon, Eun-Jung Rhee, Cheol-Young Park, Ki-Won Oh, Won-Young Lee, Sung-Woo Park, Se Eun Park

**Affiliations:** ^1^ Division of Endocrinology and Metabolism, Department of Internal Medicine, Kangbuk Samsung Hospital, Sungkyunkwan University School of Medicine, Seoul, South Korea; ^2^ Division of Endocrinology and Metabolism, Department of Internal Medicine, Korea University College of Medicine, Seoul, South Korea; ^3^ Division of Biostatistics, Department of R&D Management, Kangbuk Samsung Hospital, Sungkyunkwan University School of Medicine, Seoul, South Korea

**Keywords:** heart rate variability, autonomic nervous system, sympathetic nervous system, parasympathetic nervous system, fatty liver disease (FLD)

## Abstract

**Background:**

Although autonomic imbalance is associated with an increased risk for metabolic disease, its effects on nonalcoholic fatty liver disease (NAFLD) remains unclear. We aimed to evaluate whether autonomic dysfunction predicts the risk for nonalcoholic fatty liver disease (NAFLD).

**Methods:**

A total of 33,899 participants without NAFLD who underwent health screening programs between 2011 and 2018 were enrolled. NAFLD was identified by ultrasonography. Autonomic activity was estimated using heart rate variability (HRV). Time domain [standard deviation of the normal-to-normal interval (SDNN) and root mean square difference (RMSSD)]; frequency domain [total power (TP), low frequency (LF), and high frequency (HF), and LF/HF ratio were analyzed.

**Findings:**

A total 6,466 participants developed NAFLD within a median of 5.7 years. Subjects with incident NAFLD showed decreased overall autonomic modulation and vagal activity with lowered SDNN, RMSSD, HF, normalized HF, compared to those without NAFLD. As the SDNN, RMSSD, TP, LF, and HF tertiles increased, the risk of NAFLD decreased with tertile 1 being the reference group [the hazard ratios (95% confidence intervals) of tertile 3 were 0.90 (0.85–0.96), 0.83 (0.78–0.88), 0.91 (0.86-0.97), 0.93 (0.87-0.99) and 0.89 (0.83-0.94), respectively] after adjusting for potential confounders. The risk for NAFLD was significantly higher in subjects in whom sustained elevated heart rate, normalized LF, and LF/HF ratio values than in those with sustained decrease in these parameters during follow-up.

**Conclusions:**

Overall autonomic imbalance, decreased parasympathetic activity, and recently increased sympathetic activity might increase the risk of NAFLD.

## Introduction

Nonalcoholic fatty liver disease (NAFLD) is one of the most common liver diseases in modernized countries and has a rapidly increasing global prevalence (1–3). Therefore, many studies have focused on the pathogenesis and finding the predictive factors of NAFLD. Obesity, dyslipidemia, and insulin resistance have been regarded as well-known risk factors for fatty liver (4).

Recently, we provided evidence that decreased vagal activity and deviations in sympathetic activity were associated with the development of diabetes (5). Imbalance in the autonomic nervous system (ANS) as an early signal of insulin resistance and metabolic diseases has been reported to have clinical implications (6). Previous studies have shown a relationship between hepatic steatosis and autonomic dysfunction (7–10). However, these were small-sized, single-center, and cross-sectional studies.

The aim of the present study was to explore the longitudinal association between heart rate variability (HRV), which is a noninvasive measure of autonomic imbalance, and the risk for NAFLD in Asian adults. We also investigated the relationship between HRV and the possibility of advanced liver fibrosis.

## Methods

### Study Population and Design

This longitudinal cohort study comprised participants from a Kangbuk Samsung Health Study who underwent comprehensive health examinations annually or biennially at the Kangbuk Samsung Hospital Total Healthcare Centers in Seoul and Suwon, South Korea. In Korea, it is mandatory to participate in regular health check-up programs for every employee and companies to enhance the early detection of disease (11). For this study, we selected subjects who underwent health checkup, aged ≥18 years, including abdominal ultrasonography and HRV measurements, at least twice between January 1, 2011 and December 31, 2018 (**Supplementary Figure 1**). The initial study population comprised 80,784 subjects. We excluded subjects with fatty liver confirmed by abdominal ultrasonography upon the initial examination (n = 13,281) and positive hepatitis viral serology at the baseline (n = 681); male and female participants with alcohol consumption of >30 g/day and 20 g/day, respectively (n = 7,957); and subjects who self-reported a history of hepatitis and/or liver disease (n = 9,027). To minimize the influence of chronic diseases and medications on HRV, participants who had any of the following conditions at baseline were excluded: age of >65 years (n = 48); anemia (n = 81); estimated glomerular filtration rate (eGFR) <60 mL/min/1.73 m^2^ (n = 84); serum C-reactive protein (CRP) level >1.0 mg/dL (n = 3); abnormal thyroid function and history of thyroid disease with the related medications (n = 6,012); and history of chronic obstructive pulmonary disease and/or bronchial asthma (n = 1,101), heart disease (n = 351), history of hypertension and/or intake of any antihypertensive agent or individuals with abnormal blood pressure (systolic/diastolic blood pressure of 140/90 mmHg or higher) (n = 4,796), and malignancy (n = 1,361). Heart disease was defined as angina, myocardial infarction, or arrhythmia that needed treatment. Subjects with any missing data were excluded (n = 3,223). Total 33,899 participants were included in final analyses.

All participants provided written informed consent for the use of their data in this study. This study was approved by the institutional review board of Kangbuk Samsung Hospital (IRB No. KBS12089).

### Anthropometric and Laboratory Measurements

Data on medical history, medication use, and health-related behaviors were collected through a self-administered questionnaire. Physical measurements and serum biochemical parameters were obtained by trained staff after 12 hours of fasting. The questionnaire was based on the fourth Korea National Health and Nutritional Examination Surveys (12) and the Korean version of International Physical Activity Questionnaire short form (13). Body mass index (BMI) was defined as body weight in kilograms divided by height in meters squared. A BMI cutoff of 25 kg/m2 was used to define obesity in this Korean study population. Systolic and diastolic blood pressures (BPs) were measured with a standardized sphygmomanometer.

Glycated hemoglobin (HbA1c), fasting plasma glucose (FPG), aspartate aminotransferase (AST), alanine aminotransferase (ALT), total cholesterol, triglyceride, high-density lipoprotein cholesterol(HDL-C), and low-density lipoprotein cholesterol (LDL-C) levels were measured after 12 hours of fasting. Dyslipidemia was defined as total cholesterol level of ≥240 mg/dL (≥6.21 mmol/L) or intake of antihyperlipidemic medications. Diabetes was defined as FPG level ≥126 mg/dL, HbA1c level ≥6.5%, and/or current use of antihyperglycemic medications (14). eGFR was calculated using the Chronic Kidney Disease Epidemiology Collaboration equation (15). Serum hs-CRP level was analyzed by a BNII nephelometer (Dade Behring, Deerfield, IL, USA). Insulin resistance was assessed with the homeostatic model assessment–insulin resistance (HOMA-IR) equation (16).

### Measurement of Heart Rate Variability

As a component of the health screening exam, HRV was measured by three-minute recordings using a 3000P analyzer (Medicore Co., Ltd., Seoul, Korea), while the subject was seated in a quiet room. Participants were asked to stay still with their eyes open, remain silent, and breathe normally during the procedure. According to the methodological standards recommended by the Task Force of the European Society of Cardiology and the North American Society of Pacing and Electrophysiology for Heart Rate Variability, HRV was analyzed in both the time and frequency domains (17).

The time domain parameters included standard deviation of the normal-to-normal interval (SDNN; ms) and the square root of the mean squared difference of successive RR intervals.

### Diagnosis of Nonalcoholic Fatty Liver Disease and Advanced Liver Fibrosis

(RMSSD; ms). SDNN is a marker of overall autonomic modulation, whereas RMSSD reflects cardiac parasympathetic drive (18). For the spectral (frequency) domain, total power (TP) (0–0.4 Hz, ms2), low frequency (LF) (0.04–0.15 Hz, ms2), and high frequency (HF) (0.15–0.4 Hz, ms2) were analyzed. LF is generally considered an index of both sympathetic and parasympathetic activities, whereas HF represents parasympathetic activity in the sinus node. Normalized LF norm and HF norm were calculated from the LF/(LF + HF) and HF/(LF + HF) percentile units, with emphasis on changes in sympathetic and parasympathetic (vagal) regulation, respectively. Moreover, the LF/HF ratio, which represents sympathovagal balance was calculated (19).

The study endpoint was development of the first NAFLD diagnosis by abdominal ultrasonography (Logic Q700 MR; GE, Milwaukee, WI, USA) or December 31, 2018. NAFLD was identified based on known standard criteria, including hepatorenal echo contrast, liver brightness, and vascular blurring using a 3.5-MHz probe. Eleven experienced radiologists who were unaware of this study and were blinded to the clinical status of the subjects performed the ultrasound examinations. The inter- and intraobserver reliabilities for fatty liver diagnosis were high, with kappa statistics of 0.74 and 0.94, respectively (20). The follow-up period was designated as the time from the baseline examination to the first diagnosis of NAFLD or December 31, 2018. As a surrogate marker for advanced liver fibrosis, NAFLD Fibrosis Score (NFS) and Fibrosis 4 (FIB 4) index were used (21, 22). NFS of less than −1.455 or FIB 4 index of less than 1.3 excluded advanced liver fibrosis with high accuracy.

### Statistical Analysis

Continuous variables were presented as mean ± standard deviation. Categorical variables were expressed as numbers (percentages). Right-skewed variables (i.e., HOMA–IR, hs-CRP, AST, ALT, alcohol intake, TSH, free T4, and free T3) underwent logarithmic transformation for analysis. Student’s t-test for continuous variables and chi-square test for categorical variables were used to compare the baseline characteristics between subjects who developed NAFLD and those who did not.

To estimate hazard ratios (HRs) and 95% confidence intervals (95% CI) for the development of NAFLD, Cox proportional hazards models were used according to tertiles of HRV variables at baseline, with tertile 1 being the reference group. Model 1 was adjusted for age, sex, body mass index, current smoking, alcohol intake, regular exercise, aspartate transaminase, low-density lipoprotein cholesterol levels, and systolic blood pressure. Model 2 was adjusted for the same parameters as model 1 plus high-sensitivity C-reactive protein, homeostasis model assessment for insulin resistance score, and presence of diabetes.

A sensitivity analysis was performed on 9,673 individuals in whom HRV examination was repeated within two years. These subjects were stratified into four groups, based on the median value of each HRV variable, as follows:

Group 0: V1 exam (baseline) < median, V2 exam (follow-up) < median: reference group;
Group 1: V1 exam < median, V2 exam ≥ median;
Group 2: V1 exam ≥ median, V2 exam < median; and
Group 3: V1 exam ≥ median, V2 exam ≥ median.

Future risk for NAFLD was explored using the above-mentioned cox analysis. In addition, subgroup analysis was conducted according to sex and the presence of diabetes or dyslipidemia.

All reported p values were two-tailed, and p values <0.05 were considered statistically significant. Bonferroni correction was performed for multiple comparisons. Statistical analyses were performed using STATA version 16.0 (StataCorp, College Station, TX, USA).

## Results

### Baseline Characteristics and HRV Indices of the Participants

During 168,413 person-years of follow-up [median (interquartile range) 5.7 (4.3–6.3) years], a total of 6,466 subjects developed NAFLD. As shown in **Table 1**, compared with the subjects in the control group, the subjects who developed NAFLD were more likely to be obese, had higher levels of liver enzymes, and had metabolically worse lipid levels. The proportion of subjects with comorbid diabetes and obesity was higher in the NAFLD group than in the control group.

**Table 1:** Baseline characteristics of participants according to development of non-alcoholic fatty liver disease.

Variable	Total (N = 33, 899)	Control (N = 27,433)	NAFLD (N = 6, 466)	*P* value^a^
Age (years)	35.6 ± 5.7	35.4 ± 5.7	36.3 ± 5.6	<0.001
Sex, Men (%)	14632 (43.2)	9953 (36.3)	4679 (72.4)	<0.001
BMI (kg/m^2^)	21.9 ± 2.7	21.5 ± 2.5	23.8 ± 2.5	<0.001
Waist circumference (cm)	77.8 ± 8	76.5 ± 7.6	83.9 ± 7.0	<0.001
FBG (mg/dL)	92.6 ± 9.5	92 ± 9	95.3 ± 10.8	<0.001
Hemoglobin A1c (%)	5.6 ± 0.3	5.5 ± 0.3	5.6 ± 0.4	<0.001
HOMA-IR	1.20 ± 0.70	1.13 ± 0.62	1.46 ± 0.91	<0.001
SBP (mmHg)	105.2 ± 11	104.1 ± 10.8	109.9 ± 10.7	<0.001
AST (IU/L)	19.2 ± 8.4	18.9 ± 8.5	20.5 ± 7.6	<0.001
ALT (IU/L)	17.1 ± 11.1	16 ± 10.3	21.8 ± 13.1	<0.001
Total cholesterol (mg/dL)	187.5 ± 31.2	185.5 ± 30.5	196.3 ± 32.4	<0.001
Triglyceride (mg/dL)	112.3 ± 28.8	109.4 ± 28	124.3 ± 29.3	<0.001
HDL-C (mg/dL)	90 ± 49	83.3 ± 41.9	118.4 ± 64.3	<0.001
LDL-C (mg/dL)	61.2 ± 14.2	62.9 ± 14.1	53.9 ± 12.2	<0.001
hs-CRP (mg/dL)	0.09 ± 0.28	0.08 ± 0.28	0.11 ± 0.30	<0.001
Hemoglobin (g/dL)	14.2 ± 1.5	14 ± 1.5	15 ± 1.5	<0.001
TSH (μIU/mL)	2.09 ± 1.003	2.10 ± 1.01	2.04 ± 0.98	<0.001
Free T4 (ng/dL)	1.3 ± 0.2	1.3 ± 0.2	1.3 ± 0.2	<0.001
Free T3 (pg/mL)	3.2 ± 0.4	3.1 ± 0.4	3.3 ± 0.4	<0.001
Alcohol intake (g/day)	6.1 ± 6.6	5.7 ± 6.3	8.1 ± 7.5	<0.001
Current smoker (%)	5042 (14.9)	3265 (11.9)	1777 (27.5)	<0.001
Regular exercise (%)^b^	4330 (12.8)	3473 (12.7)	857 (13.3)	0.073
Obesity (%)^c^	4204 (12.4)	2330 (8.5)	1874 (29)	<0.001
DM (%)	234 (0.7)	146 (0.5)	88 (1.4)	<0.001
Dyslipidemia (%)	3146 (9.3)	2184 (8)	962 (14.9)	<0.001

Data are presented as mean ± standard deviation or number (%).

AST, aspartate transaminase; ALT, alanine aminotransferase; BMI, body mass index; DM, diabetes mellitus; FBG, fasting blood glucose; HDL-C, high density lipoprotein cholesterol; HOMA-IR, homeostatic model assessment-Insulin resistance; hs-CRP, high-sensitivity c-reactive protein; HTN, hypertension; LDL-C, low-density lipoprotein cholesterol; NAFLD, non-alcoholic fatty liver disease; SBP, systolic blood pressure; TSH, thyroid-stimulating hormone.

a Student’s t-tests for continuous variables and Chi-square tests for categorical variables were used to compare characteristics of the study subjects at baseline. Right-skewed variables (HOMA-IR, AST, ALT, hs-CRP, TSH, Free T4, Free T3, and Alcohol intake) were log-transformed for Student’s t-tests.

b Regular exercise was defined as performing > 20 minutes of vigorous physical activity at least three times per week.

c BMI cutoff of 25kg/m^2^ was used to define obesity for Korean population in this study.

The HRV indices SDNN, RMSSD, HF, and HF norm were lower, whereas heart rate, LF, LF norm, and LF/HF ratio were higher in the NAFLD group than in the control group (**Table 2**). Among the participants who were grouped according to the NFS and FIB 4 index (21, 22), those who had advanced liver fibrosis according to NFS showed significantly lower SDNN, RMSSD, TP, LF, and HF, compared with the values in the participants who did not have advanced liver fibrosis. The heart rate was higher in participants with advanced liver fibrosis defined by NFS than in the control group, not reaching statistical significance. Those who had advanced liver fibrosis according to FIB 4 index, showed lower heart rate, SDNN, TP, LF, and HF compared with the values in the control group.

**Table 2:** Comparison of heart rate variability indices in participants according to development of non-alcoholic fatty liver disease and possibility of advanced liver fibrosis.

	NAFLD			NFS^b^			FIB-4^c^		
	Contro l (N = 27433)	NAFLD (N = 6466)	*P* value^a^	<-1.455 (N = 6, 372)	≥-1.455 (N = 89)^d^	*P* value^a^	<1.3 (N = 6, 293)	≥1.3 (N = 170)^e^	*P* value^a^
Heart rate	64.5 ± 8.4	65.1 ± 8.5	<0.001	65.7 ± 8.7	66.7 ± 7.6	0.206	65.7 ± 8.7	64 ± 8.5	0.009
SDNN (ms)	45.6 ± 16.6	44.8 ± 16.1	<0.001	44.5 ± 16.5	36.9 ± 13.4	<0.001	44.5 ± 16.4	41.6 ± 17.8	0.018
RMSSD (ms)	42.8 ± 19	39.6 ± 17.9	<0.001	38.9 ± 17.8	30.5 ± 12.2	<0.001	38.8 ± 17.7	36.4 ± 18.5	0.080
TP (ms^2^)	1618.2 ± 1387.4	1596.1 ± 1407.3	0.250	1596.4 ± 1450.1	1021.7 ± 866.8	<0.001	1594.1 ± 1444.1	1331.9 ± 1440.7	0.005
LF (ms^2^)	442.1 ± 579	476.1 ± 632.6	<0.001	475.8 ± 635.1	248.2 ± 245.3	<0.001	473.7 ± 624.6	413.8 ± 854.4	0.008
HF (ms^2^)	540 ± 520.3	467.5 ± 456.3	<0.001	455 ± 451.7	272.9 ± 229.4	<0.001	455 ± 451	343.9 ± 377.4	0.006
LF norm	42.3 ± 20.6	47.2 ± 20.5	<0.001	47.9 ± 20.7	46.5 ± 19.6	0.951	47.9 ± 20.6	47.2 ± 20.8	0.951
HF norm	56.9 ± 20.9	52.1 ± 20.6	<0.001	51.4 ± 20.7	51.8 ± 19.8	0.529	51.4 ± 20.7	51.8 ± 20.9	0.843
LF/HF ratio	1.2 ± 2	1.4 ± 2.1	<0.001	1.5 ± 2	1.3 ± 1.4	0.727	1.5 ± 2	1.5 ± 1.8	0.892

HF, high-frequency; HF norm, normalized high-frequency; LF, low-frequency; LF norm, normalized low-frequency; NAFLD, non-alcoholic fatty liver disease; NFS, NAFLD fibrosis score; RMSSD, root mean square difference; SDNN, standard deviation of the normal-to-normal interval; TP, total power.

a Student’s t-tests were used to compare heart rate variability indices.

b In patients with NFS < -1.455, advanced liver fibrosis can be excluded with high accuracy.

c In patients with FIB-4 < 1.3, advanced liver fibrosis can be excluded.

d Five patients with missing data during follow-up was excluded when calculating NFS.

e Three patients with missing data during follow-up was excluded when calculating FIB-4 index.

### Relationship Between HRV Indices and the Risk for NAFLD

**Figure 1:**
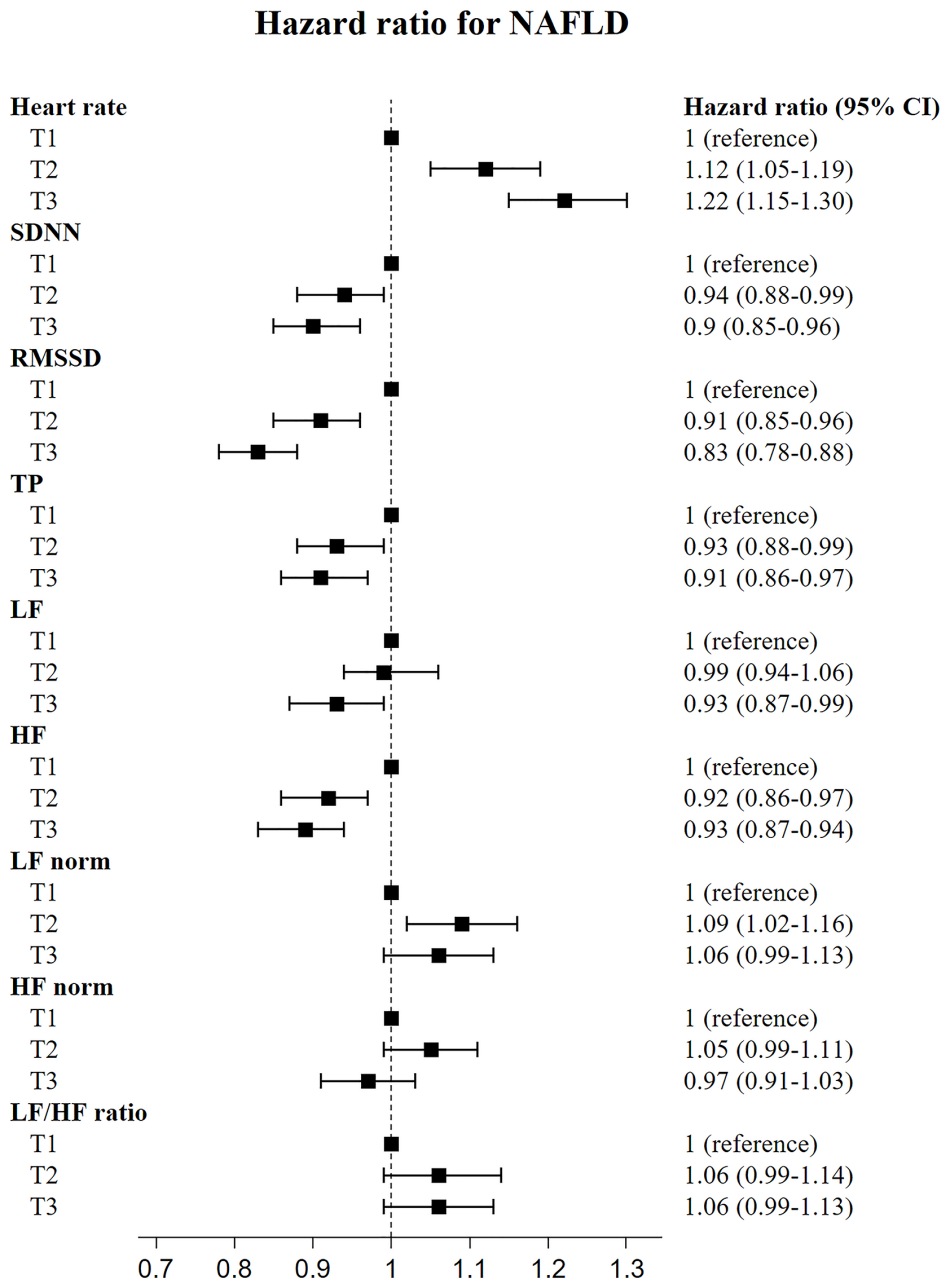
Risk of incident non-alcoholic fatty liver disease according to tertiles of heart rate variability indices.

**Figure 2:**
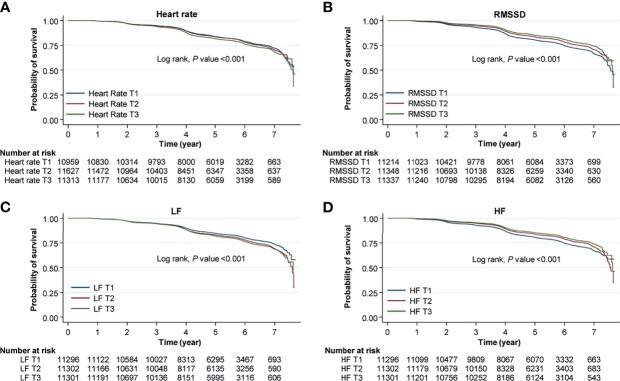
Kaplan–Meier curves for the risk for incident NAFLD according to HRV measurement. Each curves represent the risk for incident NAFLD according to tertiles of **(A)** heart rate, **(B)** root mean square difference (RMSSD), **(C)** low frequency (LF), and **(D)** high frequency (HF). Original data shown in the is included in supplemental materials (**Supplementary Table 4**).

We investigated the risk for NAFLD according to the tertile of HRV variables. As shown in **Figures 1**, **2**, the fully adjusted HRs (Model 2) of the highest tertiles of SDNN, RMSSD, TP, LF, and HF were significantly decreased, compared with those of the lowest tertiles. For heart rate, a positive relationship between each tertile and the risk for incident NAFLD was observed.

**Figure 3:**
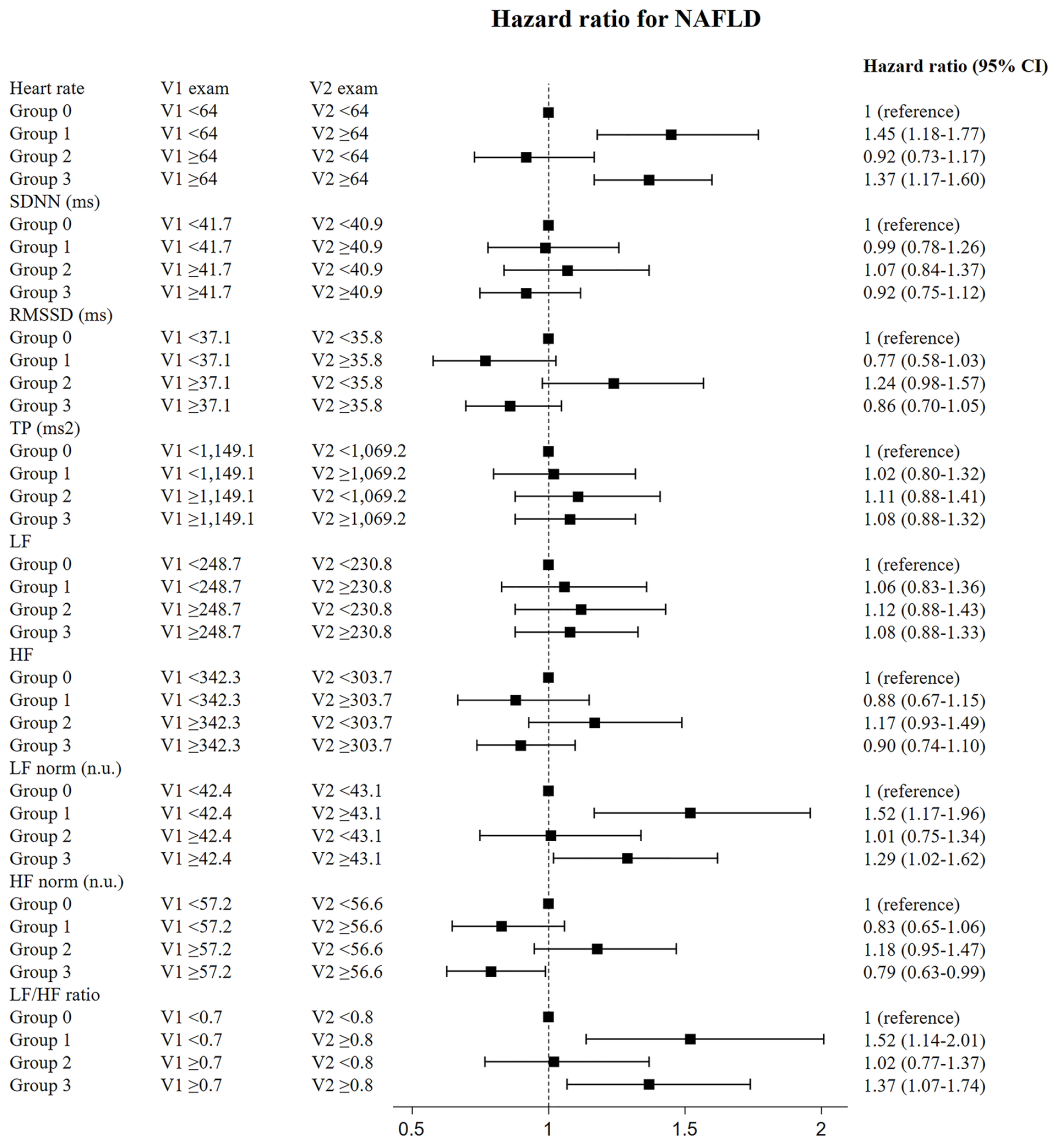
Risk of incident diabetes according to change of HRV measurement in the participants who underwent follow-up HRV exam within two years. Data shown in this figure is included in supplemental materials (**Supplementary Table 5**).

In the sensitivity analysis for subjects who had follow-up HRV measurements within two years, the risk for NAFLD was significantly higher in those who had sustained elevations in heart rate, LF norm, and LF/HF ratio (group 3) than in those who had sustained decrease in heart rate, LF norm, and LF/HF ratio (reference group, group 0); individuals who had continuously increased HF norm (group 0) exhibited lower risk (**Figure 3**). The subjects who had follow-up heart rate, LF norm, and LF/HF ratio that were newly elevated, compared with the median values (group 1), showed higher risk for NAFLD, compared with the risk of group 0. The HRs of group 1 were higher, compared with those of group 3. The risk for NAFLD group was significantly lower in those with sustained elevation of HF norm (group 3) than in group 0 (**Figure 3**). Original data shown in the **Figures 2**, **3** is included in supplemental materials (**Supplementary Tables 4**, **5**, respectively).

### Subgroup Analyses

In the subgroup analyses, there was a significant interaction between sex or presence of diabetes and the risk for incident NAFLD for all HRV indices (**Supplementary Tables 1**, **2**). In cases with dyslipidemia, incident NAFLD had a significant interaction with heart rate, SDNN, RMSSD, TP, LF, and HF (**Supplementary Table 3**). The relatively low risk for NAFLD in tertiles 3 of SDNN, RMSSD, TP, LF, and HF and the high risk in heart rate were consistently observed in men and in subjects without diabetes or dyslipidemia. In women and in subjects with diabetes and dyslipidemia, this trend was not observed, except for heart rate, RMSSD, HF (only in patients with dyslipidemia), and LF/HF ratio (only in patients with diabetes).

## Discussion

This longitudinal cohort study revealed that decreased overall autonomic function and parasympathetic activity were associated with the development of NAFLD and liver fibrosis in Asian participants, even after adjusting for potential confounders. This significance was especially observed in men and in subjects without diabetes or dyslipidemia. Also, we observed recent autonomic imbalance, especially increased sympathetic activity, might be related to the development of NAFLD.

Previous studies have suggested that autonomic imbalance might be a component in the pathogenesis of NAFLD (23, 24). In 497 Taiwanese who had health checkups, SDNN showed a negative correlation with the presence NAFLD (25). Several cross-sectional studies have suggested that the grade of NAFLD and diabetic status contributed to the decrease in cardiovascular autonomic function and overall activity (7). Consistent with these results, lower cardiovagal tone was found to be associated with hepatic steatosis in patients with recent-onset T2DM (8). In addition, a small cohort study based on magnetic resonance spectroscopy and liver biopsy reported that compared with controls, individuals with hepatic steatosis had higher proportion of autonomic dysfunction and that inflammation was independently related with autonomic dysfunction (9). However, all these previous studies were conducted with a small number of subjects and had a cross-sectional design. The findings of this present study were in line with those of previous studies mentioned above. In addition, our study extended the previous findings by investigating the longitudinal association between autonomic imbalance, based on HRV measurements, and the risk for incident NAFLD.

A diagram describing the hypothetical mechanisms of this study is shown in **Supplementary Figure 2**. The mechanisms linking autonomic dysfunction with NAFLD are not yet fully elucidated. However, there had been several laboratory findings that described the role of ANS in hepatic lipid metabolism (26). The pathophysiology of NAFLD is closely related with insulin resistance (27). Subjects with insulin resistance exhibit a defect in suppression of lipolysis in adipose tissue, resulting in increased free fatty acid efflux from adipose tissue to the liver (28). Zeng et al. (29) have shown that direct SNS innervation of adipocytes can stimulate lipolysis, thereby, possibly contributing to insulin resistance from the elevated free fatty acid levels. About 60% of liver triglycerides in patients with NAFLD are known to be derived from white adipose tissue lipolysis, which is stimulated by sympathetic activation and insulin resistance (23, 30). In rats, excessive elevations in efferent hepatic sympathetic outflow promoted hepatic steatosis, whereas hepatic sympathetic denervation reduced obesity-induced hepatic steatosis (31). On the other hand, several studies have reported anti-inflammatory properties of vagus nerve stimulation (32) and suggested that vagus nerve mediated cholinergic activation had anti-infprotective effects against obesity-related inflammation and other metabolic complications (33, 34). Nishio et al. (35) have shown that vagus nerve-mediated cholinergic signaling played a key role in regulating inflammatory responses in Kupffer cells and eventually contributed to the suppression of nonalcoholic steatohepatitis progression. In addition, the hepatic parasympathetic branch could inhibit the secretion of very low-density lipoprotein and might contribute to hepatic steatosis in the early stages of NAFLD (23, 26). Consistent with laboratory data, our study showed that decreased vagal activity and recently increased sympathetic activity might lead to the development of NAFLD. Furthermore, the degree of the fatty liver condition, which was determined by elevated markers of liver fibrosis, was relatively severe in subjects with overall autonomic imbalance and decreased vagal activity.

The results of sensitivity analysis is quite interesting. The subjects with recently elevated HR, LF norm, and LF/HF ratio, showed higher HRs for NAFLD, compared with those with sustained elevated HR, LF norm and LF/HF ratio. Recent deviation in sympathovagal imbalance to sympathetic activity and decreased parasympathetic activity might be related to increased risk of developing NAFLD. To our knowledge, no studies have investigated whether recent alterations in HRV variables have different effects on clinical outcomes than sustained alterations in HRV variables.

In the subgroup analysis by sex, the more significant findings in men than in women might be explained by the higher incidence rate of NAFLD in men than in women and the suggested susceptibility of men to autonomic dysfunction (36). Prior studies reported that sex difference in HRV indices correlated with insulin resistance and diabetic neuropathy (36, 37). Interestingly, compared with individuals without diabetes or dyslipidemia, those with diabetes or dyslipidemia had less association with incident NAFLD, despite the higher incidence. Either hyperglycemia or dyslipidemia, might have played more critical roles in the development of NAFLD rather than autonomic imbalance (7).

This present study had several distinguishing features. To the best of our knowledge, this was the first longitudinal study that examined the association between autonomic imbalance and incident NAFLD. Our robust investigation with a relatively large sample and a long follow-up period provided strong evidence that overall autonomic imbalance, decreased parasympathetic activity, and recently increased sympathetic activity might predict the risk for incident NAFLD. Moreover, we assessed the relationship between noninvasive indices of hepatic fibrosis and autonomic dysfunction.

However, this study had several limitations. First, fatty liver was diagnosed by abdominal ultrasonography not by liver biopsy. Therefore, we could not assess the severity of fatty liver in this study. Although liver biopsy is the gold standard method for the diagnosis of fatty liver, it is invasive and unsuitable as a screening tool, especially in a large-scale cohort study. On the other hand, diagnosis of fatty liver by ultrasonography has been widely used both clinically and in population-based studies because of its noninvasive nature and acceptable degree of diagnostic accuracy for steatosis (38). Second, we conducted three-minute not five-minute HRV measurements, and we did not perform repeated measurements during the health checkup program because of the standardized short-term HRV measurement protocols (17). Nevertheless, a previous study demonstrated that three minutes was the required minimal recording period that would correlate with five-minute HRV measurements (39). Regarding information on the physical activity, history of comorbidities and medication was based on a self-reported questionnaire, which is subject to recall response bias. Regarding the issue of implementation of repeated HRV measurements, we showed consistent results for individuals who had prospective follow-up HRV tests within two years. Finally, our results were derived from a sample of relatively healthy young and middle-aged educated Koreans who participated in a health checkup program regularly. Those who regularly receive a health checkup are likely to have healthier lifestyles involving good diets. We included smoking status, alcohol consumption and levels of physical activity in regression models but diet is another important factor related with pathogenesis of NAFLD; therefore, the lack of data regarding calorie intake and diet is another limitation of our study. Therefore, generalization of our results to other ethnicities or age groups might be limited. Additional studies should be performed in multiple institutes and on different ethnicities in the future.

Although the causal relationship between altered HRV and risk for fatty liver development could not be fully addressed in the present study, we observed that autonomic imbalance preceded incident NAFLD. These findings have clinical implications, possibly extending the utility of HRV measurement for risk assessment of NAFLD. Given the complexity of the pathophysiology of NAFLD, no pharmacological agents has been approved by regulatory authorities and organizations. At present, lifestyle modifications including weight reduction is recommended for prevention and treatment of NAFLD. There are increasing numbers of researches reporting the risk of various diseases and mortality among patients with NAFLD, even with mild, early stages of the disease (40–42). Therefore, detecting the alterations in HRV in advance or at patients’ earlier stages of NAFLD would be helpful for clinicians identifying patients who need interventions such as lifestyle modifications. Though additional studies are needed to clarify the roles of autonomic imbalance in the development of NAFLD, our study further suggests a new perspective for therapeutic potential of bioelectrical medicine for NAFLD.

## Conclusions

Autonomic imbalance was associated with an increased risk for incident NAFLD. In addition, decreased parasympathetic activity or recently increased sympathetic activity increased the risk for the development of NAFLD. These findings suggested that ANS impairment might play a role in the pathogenesis of NAFLD.

## Data Availability Statement

The data analyzed in this study is subject to the following licenses/restrictions: The original dataset in this study are available from Kangbuk Samsung Health Study database. The availability of these data, which were used under approval for this current study, and so are not publicly available. Requests to access these datasets should be directed to sh703.yoo@samsung.com.

## Ethics Statement

This study was approved by the Institutional Review Board of Kangbuk Samsung Hospital of Korea (IRB No. KBS12089). Participants who underwent national health check-up examinations provided written informed consent for the use of their data for research purposes. All personal information was deleted and only non-identifiable data were included for analysis.

## Author Contributions

IJ and DYL wrote and edited the original draft and did formal analysis. MYL performed data curation and investigation. HK performed data curation. E-JR supervised the study design. C-YP performed data curation. K-WO revised the manuscript critically for intellectual content. W-YL was in charge of methodology and did review and editing. S-WP performed data curation. SEP conceptualized the study and approved the final manuscript. All authors contributed to the article and approved the submitted version.

## Conflict of Interest

The authors declare that the research was conducted in the absence of any commercial or financial relationships that could be construed as a potential conflict of interest.

## Publisher’s Note

All claims expressed in this article are solely those of the authors and do not necessarily represent those of their affiliated organizations, or those of the publisher, the editors and the reviewers. Any product that may be evaluated in this article, or claim that may be made by its manufacturer, is not guaranteed or endorsed by the publisher.

## Abbreviations

ANS, autonomic nervous system; BMI, body mass index; HOMA–IR, homeostasis model assessment of insulin resistance; HRV, heart rate variability; IR, insulin resistance; LDL, low-density lipoprotein; NAFLD, nonalcoholic fatty liver disease; SNS, sympathetic nervous system; T2DM, type 2 diabetes mellitus; TG, triglyceride.
