# Supplementary material for: Autonomic Imbalance Increases the Risk for Non-alcoholic Fatty Liver Disease
Source: Front Endocrinol (Lausanne). 2021 Nov 8;12:752944. doi: 10.3389/fendo.2021.752944 (PMC8606663; doi:10.3389/fendo.2021.752944)
Supplement: Supplementary file 1 [file DataSheet_1.docx]

Supplementary Material

# Supplementary Figures

**
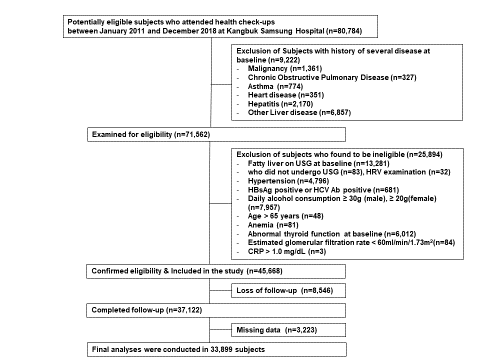
**

**Supplementary Figure 1** The figure shows the process used to select study participants for analysis. Abbreviations: N, number of individuals; USG, ultrasonography.

**
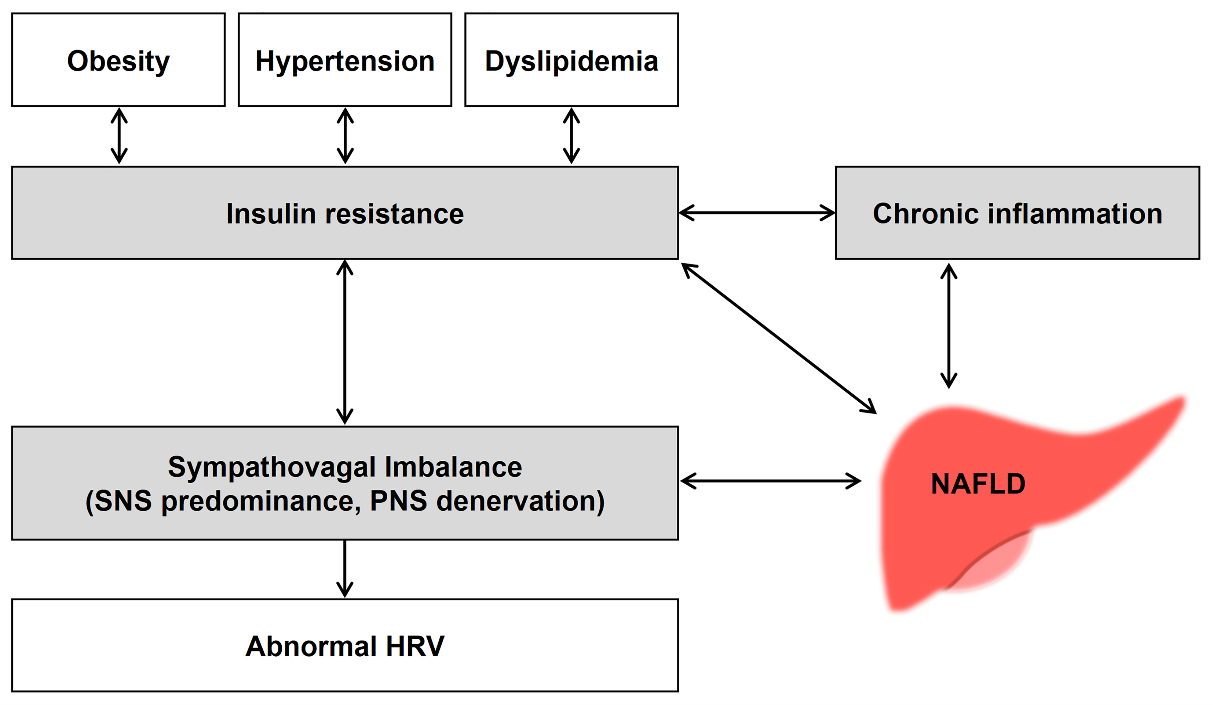
**

**Supplementary Figure 2** Causal diagram describing the hypothesis of this study. Abbreviations: HRV, heart rate variability; NAFLD, nonalcoholic fatty liver disease; SNS, sympathetic nervous system; PNS, parasympathetic nervous system.

# Supplementary Tables

**Supplementary Table 1** Risk of incident NAFLD according to tertile of HRV measurement stratified by sex.

| **Variable** | **Person-time** | **Number**  **of events** | **Incidence Rate (100 person-years) (95% CI)** | **Age-adjusted HR (95% CI)^a^** | **Multivariate-adjusted HR (95% CI)^a^** | |
| --- | --- | --- | --- | --- | --- | --- |
|  |  |  |  |  | **Model 1^b^** | **Model 2^c^** |
| Heart rate |  |  |  |  |  |  |
| *P* for interaction |  |  |  | <0.001 | <0.001 | <0.001 |
| Women |  |  |  |  |  |  |
| T1 | 26761.6 | 350 | 1.31 (1.18-1.45) | 1 (reference) | 1 (reference) | 1 (reference) |
| T2 | 34992.4 | 624 | 1.78 (1.65-1.93) | 1.37 (1.20-1.56) | 1.24 (1.08-1.41) | 1.23 (1.07-1.40) |
| T3 | 35771.4 | 813 | 2.27 (2.12-2.43) | 1.74 (1.54-1.98) | 1.32 (1.16-1.50) | 1.28 (1.12-1.46) |
| *P* for trend |  |  |  | <0.001 | <0.001 | <0.001 |
| Men |  |  |  |  |  |  |
| T1 | 27953.1 | 1647 | 5.89 (5.61-6.18) | 1 (reference) | 1 (reference) | 1 (reference) |
| T2 | 22849.8 | 1539 | 6.74 (6.41-7.08) | 1.14 (1.07-1.23) | 1.12 (1.04-1.20) | 1.08 (1.01-1.16) |
| T3 | 20084.9 | 1493 | 7.43 (7.07-7.82) | 1.27 (1.19-1.36) | 1.25 (1.16-1.34) | 1.17 (1.09-1.26) |
| *P* for trend |  |  |  | <0.001 | <0.001 | <0.001 |
| SDNN, ms |  |  |  |  |  |  |
| *P* for interaction |  |  |  | <0.001 | <0.001 | <0.001 |
| Women |  |  |  |  |  |  |
| T1 | 34209.6 | 723 | 2.11 (1.96-2.27) | 1 (reference) | 1 (reference) | 1 (reference) |
| T2 | 32813.1 | 578 | 1.76 (1.62-1.91) | 0.84 (0.75-0.94) | 0.93 (0.83-1.03) | 0.95 (0.85-1.06) |
| T3 | 30502.8 | 486 | 1.59 (1.46-1.74) | 0.77 (0.69-0.87) | 0.87 (0.77-0.98) | 0.89 (0.79-1.01) |
| *P* for trend |  |  |  | <0.001 | 0.019 | 0.062 |
| Men |  |  |  |  |  |  |
| T1 | 21923.8 | 1531 | 6.98 (6.64-7.34) | 1 (reference) | 1 (reference) | 1 (reference) |
| T2 | 23459.7 | 1535 | 6.54 (6.22-6.88) | 0.90 (0.84-0.96) | 0.92 (0.86-0.99) | 0.94 (0.87-1.01) |
| T3 | 25504.3 | 1613 | 6.32 (6.02-6.64) | 0.85 (0.79-0.91) | 0.88 (0.82-0.94) | 0.91 (0.85-0.98) |
| *P* for trend |  |  |  | <0.001 | <0.001 | 0.010 |
| RMSSD, ms |  |  |  |  |  |  |
| *P* for interaction |  |  |  | <0.001 | <0.001 | <0.001 |
| Women |  |  |  |  |  |  |
| T1 | 30403.3 | 672 | 2.21 (2.05-2.38) | 1 (reference) | 1 (reference) | 1 (reference) |
| T2 | 32586.4 | 582 | 1.79 (1.65-1.94) | 0.82 (0.73-0.91) | 0.9 (0.8-1.003) | 0.92 (0.82-1.03) |
| T3 | 34535.9 | 533 | 1.54 (1.42-1.68) | 0.71 (0.64-0.80) | 0.84 (0.75-0.95) | 0.87 (0.77-0.98) |
| *P* for trend |  |  |  | <0.001 | 0.004 | 0.022 |
| Men |  |  |  |  |  |  |
| T1 | 25052.2 | 1830 | 7.30 (6.98-7.65) | 1 (reference) | 1 (reference) | 1 (reference) |
| T2 | 24064 | 1552 | 6.45 (6.14-6.78) | 0.84 (0.79-0.9) | 0.88 (0.82-0.94) | 0.90 (0.84-0.96) |
| T3 | 21771.5 | 1297 | 5.96 (5.64-6.29) | 0.75 (0.7-0.81) | 0.78 (0.73-0.84) | 0.82 (0.76-0.88) |
| *P* for trend |  |  |  | <0.001 | <0.001 | <0.001 |
| TP, ms^2^ |  |  |  |  |  |  |
| *P* for interaction |  |  |  | <0.001 | <0.001 | <0.001 |
| Women |  |  |  |  |  |  |
| T1 | 33949.2 | 698 | 2.06 (1.91-2.21) | 1 (reference) | 1 (reference) | 1 (reference) |
| T2 | 33150.1 | 614 | 1.85 (1.71-2.00) | 0.90 (0.81-1.01) | 0.96 (0.86-1.08) | 0.99 (0.88-1.10) |
| T3 | 30426.2 | 475 | 1.56 (1.43-1.71) | 0.77 (0.69-0.87) | 0.87 (0.77-0.98) | 0.89 (0.79-1.01) |
| *P* for trend |  |  |  | <0.001 | 0.029 | 0.078 |
| Men |  |  |  |  |  |  |
| T1 | 22118.7 | 1545 | 6.99 (6.65-7.34) | 1 (reference) | 1 (reference) | 1 (reference) |
| T2 | 23364.3 | 1507 | 6.45 (6.13-6.78) | 0.89 (0.83-0.96) | 0.9 (0.84-0.97) | 0.92 (0.85-0.98) |
| T3 | 25404.8 | 1627 | 6.40 (6.10-6.72) | 0.86 (0.80-0.93) | 0.9 (0.84-0.96) | 0.92 (0.86-0.99) |
| *P* for trend |  |  |  | <0.001 | 0.003 | 0.025 |
| LF |  |  |  |  |  |  |
| *P* for interaction |  |  |  | <0.001 | <0.001 | <0.001 |
| Women |  |  |  |  |  |  |
| T1 | 38164.5 | 731 | 1.92 (1.78-2.06) | 1 (reference) | 1 (reference) | 1 (reference) |
| T2 | 32412.9 | 616 | 1.90 (1.76-2.06) | 1.01 (0.91-1.12) | 0.98 (0.88-1.09) | 0.99 (0.89-1.11) |
| T3 | 26948.1 | 440 | 1.63 (1.49-1.79) | 0.87 (0.78-0.98) | 0.91 (0.80-1.02) | 0.92 (0.82-1.04) |
| *P* for trend |  |  |  | 0.041 | 0.122 | 0.216 |
| Men |  |  |  |  |  |  |
| T1 | 18372.3 | 1238 | 6.74 (6.37-7.12) | 1 (reference) | 1 (reference) | 1 (reference) |
| T2 | 23592.3 | 1587 | 6.73 (6.40-7.07) | 0.96 (0.89-1.03) | 0.98 (0.91-1.05) | 0.99 (0.92-1.06) |
| T3 | 28923.3 | 1854 | 6.41 (6.12-6.71) | 0.89 (0.83-0.96) | 0.91 (0.85-0.98) | 0.93 (0.86-0.99) |
| *P* for trend |  |  |  | 0.002 | 0.009 | 0.034 |
| HF |  |  |  |  |  |  |
| *P* for interaction |  |  |  | <0.001 | <0.001 | <0.001 |
| Women |  |  |  |  |  |  |
| T1 | 29126.5 | 617 | 2.12 (1.96-2.29) | 1 (reference) | 1 (reference) | 1 (reference) |
| T2 | 32680.2 | 597 | 1.83 (1.69-1.98) | 0.86 (0.77-0.96) | 0.93 (0.83-1.05) | 0.96 (0.85-1.08) |
| T3 | 35718.7 | 573 | 1.60 (1.48-1.74) | 0.77 (0.68-0.86) | 0.89 (0.79-1.01) | 0.92 (0.82-1.03) |
| *P* for trend |  |  |  | <0.001 | 0.064 | 0.163 |
| Men |  |  |  |  |  |  |
| T1 | 26475.4 | 1879 | 7.10 (6.78-7.43) | 1 (reference) | 1 (reference) | 1 (reference) |
| T2 | 23995.4 | 1528 | 6.37 (6.06-6.70) | 0.85 (0.79-0.91) | 0.88 (0.82-0.95) | 0.90 (0.84-0.96) |
| T3 | 20417 | 1272 | 6.23 (5.90-6.58) | 0.80 (0.75-0.87) | 0.85 (0.79-0.91) | 0.88 (0.82-0.95) |
| *P* for trend |  |  |  | <0.001 | <0.001 | <0.001 |
| LFnorm |  |  |  |  |  |  |
| *P* for interaction |  |  |  | <0.001 | <0.001 | <0.001 |
| Women |  |  |  |  |  |  |
| T1 | 41484.7 | 699 | 1.68 (1.56-1.81) | 1 (reference) | 1 (reference) | 1 (reference) |
| T2 | 32223.4 | 600 | 1.86 (1.72-2.02) | 1.11 (0.99-1.24) | 1.05 (0.94-1.17) | 1.04 (0.93-1.17) |
| T3 | 23817.4 | 488 | 2.05 (1.87-2.24) | 1.23 (1.09-1.38) | 1.04 (0.92-1.17) | 1.01 (0.90-1.14) |
| *P* for trend |  |  |  | <0.001 | 0.479 | 0.784 |
| Men |  |  |  |  |  |  |
| T1 | 15302.8 | 938 | 6.13 (5.75-6.53) | 1 (reference) | 1 (reference) | 1 (reference) |
| T2 | 23885.6 | 1595 | 6.68 (6.36-7.01) | 1.10 (1.01-1.19) | 1.11 (1.02-1.2) | 1.10 (1.01-1.19) |
| T3 | 31699.4 | 2146 | 6.77 (6.49-7.06) | 1.12 (1.04-1.21) | 1.09 (1.01-1.17) | 1.07 (0.99-1.15) |
| *P* for trend |  |  |  | 0.006 | 0.084 | 0.207 |
| HFnorm |  |  |  |  |  |  |
| *P* for interaction |  |  |  | <0.001 | <0.001 | <0.001 |
| Women |  |  |  |  |  |  |
| T1 | 23837.6 | 471 | 1.98 (1.81-2.16) | 1 (reference) | 1 (reference) | 1 (reference) |
| T2 | 32008.7 | 601 | 1.88 (1.73-2.03) | 0.95 (0.84-1.07) | 1.05 (0.93-1.19) | 1.07 (0.94-1.21) |
| T3 | 41679.2 | 715 | 1.72 (1.59-1.85) | 0.86 (0.77-0.97) | 1.01 (0.89-1.14) | 1.04 (0.92-1.17) |
| *P* for trend |  |  |  | 0.010 | 0.961 | 0.653 |
| Men |  |  |  |  |  |  |
| T1 | 31577 | 2132 | 6.75 (6.47-7.04) | 1 (reference) | 1 (reference) | 1 (reference) |
| T2 | 23986.8 | 1608 | 6.70 (6.38-7.04) | 0.99 (0.93-1.06) | 1.03 (0.97-1.10) | 1.04 (0.98-1.11) |
| T3 | 15324.1 | 939 | 6.13 (5.75-6.53) | 0.89 (0.83-0.96) | 0.93 (0.86-1.00) | 0.94 (0.87-1.02) |
| *P* for trend |  |  |  | 0.008 | 0.155 | 0.335 |
| LF/HF ratio |  |  |  |  |  |  |
| *P* for interaction |  |  |  | <0.001 | <0.001 | <0.001 |
| Women |  |  |  |  |  |  |
| T1 | 39258.1 | 661 | 1.68 (1.56-1.82) | 1 (reference) | 1 (reference) | 1 (reference) |
| T2 | 32868.8 | 608 | 1.85 (1.71-2.00) | 1.10 (0.99-1.23) | 1.05 (0.94-1.17) | 1.04 (0.93-1.16) |
| T3 | 25398.6 | 518 | 2.04 (1.87-2.22) | 1.23 (1.09-1.37) | 1.04 (0.93-1.18) | 1.02 (0.90-1.15) |
| *P* for trend |  |  |  | 0.001 | 0.444 | 0.725 |
| Men |  |  |  |  |  |  |
| T1 | 14101.8 | 869 | 6.16 (5.77-6.59) | 1 (reference) | 1 (reference) | 1 (reference) |
| T2 | 23735 | 1563 | 6.59 (6.27-6.92) | 1.07 (0.99-1.17) | 1.07 (0.99-1.17) | 1.07 (0.98-1.16) |
| T3 | 33051 | 2247 | 6.80 (6.52-7.09) | 1.12 (1.03-1.21) | 1.08 (1.00-1.17) | 1.06 (0.98-1.15) |
| *P* for trend |  |  |  | 0.005 | 0.079 | 0.199 |

Abbreviations: CI, confidence interval; HF, high-frequency; HF norm, normalized high-frequency; HR, hazard ratio; LF, low-frequency; LF norm, normalized low-frequency; RMSSD, root mean square difference; SDNN, standard deviation of the normal-to-normal interval; TP, total power.

^a^ Cox proportional hazards regression models were used to estimate HRs and 95% CIs.

^b^ Model 1 was adjusted for age, body mass index, current smoking, alcohol intake, regular exercise, aspartate transaminase, low-density lipoprotein cholesterol levels, and systolic blood pressure.

^c^ Model 2 was adjusted for the same factors as model 1 plus high-sensitivity C-reactive protein, homeostasis model assessment for insulin resistance score, and presence of diabetes.

**Supplementary Table 2** Risk of incident NAFLD according to tertile of HRV measurement stratified by presence of diabetes.

|  | **Person-time** | **Number of events** | **Incidence Rate (1,000 person-years) (95% CI)** | **Age- and sex-adjusted HR (95% CI)^a^** | **Multivariate-adjusted HR (95% CI)^a^** | |
| --- | --- | --- | --- | --- | --- | --- |
|  |  |  |  |  | **Model 1^b^** | **Model 2^c^** |
| Heart rate |  |  |  |  |  |  |
| *P* for interaction |  |  |  | <0.001 | <0.001 | <0.001 |
| Without DM |  |  |  |  |  |  |
| T1 | 54354.6 | 1977 | 3.64 (3.48-3.8) | 1 (reference) | 1 (reference) | 1 (reference) |
| T2 | 57552.6 | 2125 | 3.69 (3.54-3.85) | 1.17 (1.1-1.25) | 1.13 (1.07-1.21) | 1.1 (1.03-1.17) |
| T3 | 55383.3 | 2261 | 4.08 (3.92-4.25) | 1.37 (1.29-1.46) | 1.27 (1.2-1.35) | 1.2 (1.12-1.27) |
| *P* for trend |  |  |  | <0.001 | <0.001 | <0.001 |
| With DM |  |  |  |  |  |  |
| T1 | 360.2 | 20 | 5.55 (3.58-8.61) | 1 (reference) | 1 (reference) | 1 (reference) |
| T2 | 289.7 | 38 | 13.12 (9.55-18.03) | 2.49 (1.45-4.29) | 2.28 (1.3-3.99) | 2.28 (1.3-4) |
| T3 | 473 | 45 | 9.51 (7.1-12.74) | 1.88 (1.1-3.21) | 1.83 (1.04-3.21) | 1.85 (1.05-3.26) |
| *P* for trend |  |  |  | 0.042 | 0.064 | 0.059 |
| SDNN, ms |  |  |  |  |  |  |
| *P* for interaction |  |  |  | <0.001 | <0.001 | <0.001 |
| Without DM |  |  |  |  |  |  |
| T1 | 55584 | 2198 | 3.95 (3.79-4.12) | 1 (reference) | 1 (reference) | 1 (reference) |
| T2 | 55919.7 | 2086 | 3.73 (3.57-3.89) | 0.89 (0.83-0.94) | 0.92 (0.87-0.98) | 0.94 (0.89-1.002) |
| T3 | 55786.8 | 2079 | 3.73 (3.57-3.89) | 0.84 (0.79-0.89) | 0.88 (0.83-0.93) | 0.91 (0.85-0.97) |
| *P* for trend |  |  |  | <0.001 | <0.001 | 0.002 |
| With DM |  |  |  |  |  |  |
| T1 | 549.3 | 56 | 10.19 (7.85-13.25) | 1 (reference) | 1 (reference) | 1 (reference) |
| T2 | 353.2 | 27 | 7.64 (5.24-11.15) | 0.67 (0.42-1.07) | 0.71 (0.44-1.15) | 0.7 (0.43-1.13) |
| T3 | 220.3 | 20 | 9.08 (5.86-14.07) | 0.79 (0.47-1.32) | 0.83 (0.48-1.42) | 0.84 (0.49-1.45) |
| *P* for trend |  |  |  | 0.218 | 0.343 | 0.361 |
| RMSSD, ms |  |  |  |  |  |  |
| *P* for interaction |  |  |  | <0.001 | <0.001 | <0.001 |
| Without DM |  |  |  |  |  |  |
| T1 | 54865.9 | 2439 | 4.45 (4.27-4.63) | 1 (reference) | 1 (reference) | 1 (reference) |
| T2 | 56298 | 2105 | 3.74 (3.58-3.9) | 0.84 (0.79-0.89) | 0.89 (0.84-0.95) | 0.92 (0.86-0.97) |
| T3 | 56126.5 | 1819 | 3.24 (3.1-3.39) | 0.75 (0.71-0.8) | 0.8 (0.75-0.85) | 0.84 (0.79-0.9) |
| *P* for trend |  |  |  | <0.001 | <0.001 | <0.001 |
| With DM |  |  |  |  |  |  |
| T1 | 589.5 | 63 | 10.69 (8.35-13.68) | 1 (reference) | 1 (reference) | 1 (reference) |
| T2 | 352.4 | 29 | 8.23 (5.72-11.84) | 0.69 (0.44-1.08) | 0.68 (0.42-1.08) | 0.67 (0.42-1.08) |
| T3 | 180.9 | 11 | 6.08 (3.37-10.98) | 0.5 (0.26-0.95) | 0.5 (0.26-0.97) | 0.5 (0.26-0.96) |
| *P* for trend |  |  |  | 0.016 | 0.020 | 0.018 |
| TP, ms^2^ |  |  |  |  |  |  |
| *P* for interaction |  |  |  | <0.001 | <0.001 | <0.001 |
| Without DM |  |  |  |  |  |  |
| T1 | 55546 | 2188 | 3.94 (3.78-4.11) | 1 (reference) | 1 (reference) | 1 (reference) |
| T2 | 56162.1 | 2094 | 3.73 (3.57-3.89) | 0.9 (0.85-0.95) | 0.92 (0.87-0.98) | 0.94 (0.88-0.999) |
| T3 | 55582.4 | 2081 | 3.74 (3.59-3.91) | 0.84 (0.79-0.9) | 0.9 (0.84-0.95) | 0.92 (0.87-0.98) |
| *P* for trend |  |  |  | <0.001 | 0.001 | 0.009 |
| With DM |  |  |  |  |  |  |
| T1 | 521.9 | 55 | 10.54 (8.09-13.73) | 1 (reference) | 1 (reference) | 1 (reference) |
| T2 | 352.3 | 27 | 7.66 (5.26-11.17) | 0.67 (0.42-1.07) | 0.71 (0.44-1.14) | 0.68 (0.42-1.1) |
| T3 | 248.6 | 21 | 8.45 (5.51-12.96) | 0.72 (0.43-1.2) | 0.71 (0.42-1.21) | 0.73 (0.43-1.23) |
| *P* for trend |  |  |  | 0.125 | 0.142 | 0.149 |
| LF |  |  |  |  |  |  |
| *P* for interaction |  |  |  | <0.001 | <0.001 | <0.001 |
| Without DM |  |  |  |  |  |  |
| T1 | 56026.5 | 1929 | 3.44 (3.29-3.6) | 1 (reference) | 1 (reference) | 1 (reference) |
| T2 | 55670.3 | 2161 | 3.88 (3.72-4.05) | 0.97 (0.91-1.03) | 0.98 (0.92-1.04) | 0.98 (0.92-1.05) |
| T3 | 55593.7 | 2273 | 4.09 (3.92-4.26) | 0.89 (0.84-0.95) | 0.91 (0.86-0.97) | 0.93 (0.87-0.99) |
| *P* for trend |  |  |  | <0.001 | 0.004 | 0.015 |
| With DM |  |  |  |  |  |  |
| T1 | 510.3 | 40 | 7.84 (5.75-10.69) | 1 (reference) | 1 (reference) | 1 (reference) |
| T2 | 334.9 | 42 | 12.54 (9.27-16.97) | 1.56 (1.01-2.42) | 1.58 (1-2.5) | 1.55 (0.98-2.46) |
| T3 | 277.7 | 21 | 7.56 (4.93-11.6) | 0.89 (0.52-1.54) | 0.94 (0.54-1.65) | 0.95 (0.54-1.67) |
| *P* for trend |  |  |  | 0.977 | 0.879 | 0.853 |
| HF |  |  |  |  |  |  |
| *P* for interaction |  |  |  | <0.001 | <0.001 | <0.001 |
| Without DM |  |  |  |  |  |  |
| T1 | 54985.1 | 2429 | 4.42 (4.25-4.6) | 1 (reference) | 1 (reference) | 1 (reference) |
| T2 | 56334.5 | 2101 | 3.73 (3.57-3.89) | 0.86 (0.81-0.91) | 0.91 (0.85-0.96) | 0.92 (0.87-0.98) |
| T3 | 55970.9 | 1833 | 3.27 (3.13-3.43) | 0.8 (0.75-0.85) | 0.87 (0.81-0.92) | 0.9 (0.84-0.96) |
| *P* for trend |  |  |  | <0.001 | <0.001 | 0.001 |
| With DM |  |  |  |  |  |  |
| T1 | 616.8 | 67 | 10.86 (8.55-13.8) | 1 (reference) | 1 (reference) | 1 (reference) |
| T2 | 341.2 | 24 | 7.03 (4.71-10.49) | 0.64 (0.4-1.02) | 0.62 (0.38-1.01) | 0.64 (0.39-1.04) |
| T3 | 164.8 | 12 | 7.28 (4.14-12.82) | 0.6 (0.32-1.12) | 0.65 (0.35-1.22) | 0.64 (0.34-1.2) |
| *P* for trend |  |  |  | 0.037 | 0.062 | 0.060 |
| LF norm (n.u.) |  |  |  |  |  |  |
| *P* for interaction |  |  |  | <0.001 | <0.001 | <0.001 |
| Without DM |  |  |  |  |  |  |
| T1 | 56481.5 | 1618 | 2.86 (2.73-3.01) | 1 (reference) | 1 (reference) | 1 (reference) |
| T2 | 55780.3 | 2162 | 3.88 (3.72-4.04) | 1.1 (1.03-1.17) | 1.09 (1.02-1.16) | 1.07 (1.01-1.15) |
| T3 | 55028.7 | 2583 | 4.69 (4.52-4.88) | 1.14 (1.07-1.21) | 1.07 (1.003-1.14) | 1.04 (0.98-1.11) |
| *P* for trend |  |  |  | <0.001 | 0.069 | 0.273 |
| With DM |  |  |  |  |  |  |
| T1 | 306 | 19 | 6.21 (3.96-9.73) | 1 (reference) | 1 (reference) | 1 (reference) |
| T2 | 328.7 | 33 | 10.04 (7.14-14.12) | 1.72 (0.97-3.04) | 1.83 (1.01-3.3) | 1.8 (0.99-3.24) |
| T3 | 488.1 | 51 | 10.45 (7.94-13.75) | 1.72 (1.01-2.94) | 1.78 (1.01-3.12) | 1.74 (0.99-3.08) |
| *P* for trend |  |  |  | 0.066 | 0.069 | 0.082 |
| HF norm (n.u.) |  |  |  |  |  |  |
| *P* for interaction |  |  |  | <0.001 | <0.001 | <0.001 |
| Without DM |  |  |  |  |  |  |
| T1 | 54935.7 | 2554 | 4.65 (4.47-4.83) | 1 (reference) | 1 (reference) | 1 (reference) |
| T2 | 55656.8 | 2174 | 3.91 (3.75-4.07) | 0.98 (0.93-1.04) | 1.03 (0.98-1.1) | 1.04 (0.99-1.11) |
| T3 | 56698 | 1635 | 2.88 (2.75-3.03) | 0.89 (0.84-0.95) | 0.96 (0.9-1.02) | 0.98 (0.92-1.04) |
| *P* for trend |  |  |  | 0.001 | 0.257 | 0.661 |
| With DM |  |  |  |  |  |  |
| T1 | 479 | 49 | 10.23 (7.73-13.54) | 1 (reference) | 1 (reference) | 1 (reference) |
| T2 | 338.6 | 35 | 10.34 (7.42-14.4) | 1.06 (0.68-1.64) | 1.1 (0.7-1.73) | 1.1 (0.69-1.73) |
| T3 | 305.2 | 19 | 6.22 (3.97-9.76) | 0.6 (0.35-1.02) | 0.59 (0.34-1.04) | 0.6 (0.34-1.06) |
| *P* for trend |  |  |  | 0.093 | 0.111 | 0.126 |
| LF/HF ratio |  |  |  |  |  |  |
| *P* for interaction |  |  |  | <0.001 | <0.001 | <0.001 |
| Without DM |  |  |  |  |  |  |
| T1 | 53080.3 | 1514 | 2.85 (2.71-3) | 1 (reference) | 1 (reference) | 1 (reference) |
| T2 | 56258.5 | 2136 | 3.8 (3.64-3.96) | 1.08 (1.01-1.15) | 1.06 (0.99-1.14) | 1.05 (0.98-1.12) |
| T3 | 57951.7 | 2713 | 4.68 (4.51-4.86) | 1.14 (1.06-1.21) | 1.07 (1-1.14) | 1.04 (0.98-1.11) |
| *P* for trend |  |  |  | <0.001 | 0.063 | 0.246 |
| With DM |  |  |  |  |  |  |
| T1 | 279.5 | 16 | 5.72 (3.51-9.34) | 1 (reference) | 1 (reference) | 1 (reference) |
| T2 | 345.3 | 35 | 10.14 (7.28-14.12) | 1.92 (1.06-3.47) | 2 (1.07-3.72) | 1.99 (1.07-3.7) |
| T3 | 498 | 52 | 10.44 (7.96-13.7) | 1.86 (1.05-3.28) | 1.9 (1.04-3) | 1.87 (1.02-3.41) |
| *P* for trend |  |  |  | 0.055 | 0.064 | 0.076 |

Abbreviations: CI, confidence interval; HF, high-frequency; HF norm, normalized high-frequency; HR, hazard ratio; LF, low-frequency; LF norm, normalized low-frequency; RMSSD, root mean square difference; SDNN, standard deviation of the normal-to-normal interval; TP, total power.

^a^ Cox proportional hazards regression models were used to estimate HRs and 95% CIs.

^b^ Model 1 was adjusted for age, sex, body mass index, current smoking, alcohol intake, regular exercise, aspartate transaminase, low-density lipoprotein cholesterol levels, and systolic blood pressure.

^c^ Model 2 was adjusted for the same parameters as model 1 plus high-sensitivity C-reactive protein, homeostasis model assessment for insulin resistance score, and presence of diabetes.

**Supplementary Table 3** Risk of incident NAFLD according to tertile of HRV measurement stratified by presence of dyslipidemia.

|  | **Person-time** | | **Number of events** | **Incidence Rate (1,000 person-years) (95% CI)** | **Age- and sex-adjusted HR (95% CI)^a^** | **Multivariate-adjusted HR (95% CI)^a^** | |
| --- | --- | --- | --- | --- | --- | --- | --- |
|  |  |  |  |  |  | **Model 1^b^** | **Model 2^c^** |
| Heart rate | |  |  |  |  |  |  |
| *P* for interaction | |  |  |  | <0.001 | <0.001 | <0.001 |
| Without dyslipidemia | |  |  |  |  |  |  |
| T1 | | 49093 | 1630 | 3.32 (3.16-3.49) | 1 (reference) | 1 (reference) | 1 (reference) |
| T2 | | 51905.6 | 1756 | 3.38 (3.23-3.55) | 1.18 (1.11-1.27) | 1.15 (1.07-1.23) | 1.11 (1.04-1.19) |
| T3 | | 49641 | 1852 | 3.73 (3.56-3.9) | 1.38 (1.29-1.47) | 1.28 (1.2-1.37) | 1.2 (1.12-1.28) |
| *P* for trend | |  |  |  | <0.001 | <0.001 | <0.001 |
| With dyslipidemia | |  |  |  |  |  |  |
| T1 | | 5621.8 | 367 | 6.53 (5.89-7.23) | 1 (reference) | 1 (reference) | 1 (reference) |
| T2 | | 5936.7 | 407 | 6.86 (6.22-7.56) | 1.16 (1-1.33) | 1.13 (0.98-1.31) | 1.12 (0.97-1.29) |
| T3 | | 6215.3 | 454 | 7.3 (6.66-8.01) | 1.32 (1.15-1.51) | 1.27 (1.1-1.46) | 1.22 (1.06-1.41) |
| *P* for trend | |  |  |  | <0.001 | 0.001 | 0.005 |
| SDNN, ms | |  |  |  |  |  |  |
| *P* for interaction | |  |  |  | <0.001 | <0.001 | 0.011 |
| Without dyslipidemia | |  |  |  |  |  |  |
| T1 | | 49799.4 | 1798 | 3.61 (3.45-3.78) | 1 (reference) | 1 (reference) | 1 (reference) |
| T2 | | 50499 | 1723 | 3.41 (3.25-3.58) | 0.88 (0.82-0.94) | 0.91 (0.85-0.97) | 0.93 (0.87-0.99) |
| T3 | | 50341.1 | 1717 | 3.41 (3.25-3.58) | 0.82 (0.77-0.88) | 0.86 (0.8-0.92) | 0.89 (0.83-0.95) |
| *P* for trend | |  |  |  | <0.001 | <0.001 | 0.001 |
| With dyslipidemia | |  |  |  |  |  |  |
| T1 | | 6333.9 | 456 | 7.2 (6.57-7.89) | 1 (reference) | 1 (reference) | 1 (reference) |
| T2 | | 5773.8 | 390 | 6.75 (6.12-7.46) | 0.89 (0.78-1.02) | 0.94 (0.82-1.07) | 0.95 (0.83-1.09) |
| T3 | | 5666 | 382 | 6.74 (6.1-7.45) | 0.87 (0.76-0.999) | 0.92 (0.8-1.06) | 0.95 (0.83-1.1) |
| *P* for trend | |  |  |  | 0.047 | 0.256 | 0.483 |
| RMSSD, ms | |  |  |  |  |  |  |
| *P* for interaction | |  |  |  | <0.001 | <0.001 | <0.001 |
| Without dyslipidemia | |  |  |  |  |  |  |
| T1 | | 48661.2 | 1980 | 4.07 (3.89-4.25) | 1 (reference) | 1 (reference) | 1 (reference) |
| T2 | | 50730 | 1721 | 3.39 (3.24-3.56) | 0.83 (0.78-0.89) | 0.88 (0.83-0.94) | 0.91 (0.85-0.97) |
| T3 | | 51248.4 | 1537 | 3 (2.85-3.15) | 0.75 (0.7-0.8) | 0.79 (0.74-0.85) | 0.83 (0.78-0.89) |
| *P* for trend | |  |  |  | <0.001 | <0.001 | <0.001 |
| With dyslipidemia | |  |  |  |  |  |  |
| T1 | | 6794.3 | 522 | 7.68 (7.05-8.37) | 1 (reference) | 1 (reference) | 1 (reference) |
| T2 | | 5920.4 | 413 | 6.98 (6.33-7.68) | 0.86 (0.76-0.98) | 0.9 (0.78-1.02) | 0.91 (0.8-1.04) |
| T3 | | 5059.1 | 293 | 5.79 (5.17-6.49) | 0.75 (0.65-0.87) | 0.79 (0.68-0.92) | 0.83 (0.71-0.96) |
| *P* for trend | |  |  |  | <0.001 | 0.002 | 0.011 |
| TP, ms^2^ | |  |  |  |  |  |  |
| *P* for interaction | |  |  |  | <0.001 | 0.001 | 0.045 |
| Without dyslipidemia | |  |  |  |  |  |  |
| T1 | | 49753.5 | 1785 | 3.59 (3.43-3.76) | 1 (reference) | 1 (reference) | 1 (reference) |
| T2 | | 50686.7 | 1721 | 3.4 (3.24-3.56) | 0.89 (0.83-0.95) | 0.91 (0.85-0.97) | 0.92 (0.86-0.99) |
| T3 | | 50199.3 | 1732 | 3.45 (3.29-3.62) | 0.84 (0.79-0.9) | 0.88 (0.83-0.95) | 0.91 (0.85-0.97) |
| *P* for trend | |  |  |  | <0.001 | <0.001 | 0.005 |
| With dyslipidemia | |  |  |  |  |  |  |
| T1 | | 6314.4 | 458 | 7.25 (6.62-7.95) | 1 (reference) | 1 (reference) | 1 (reference) |
| T2 | | 5827.7 | 400 | 6.86 (6.22-7.57) | 0.93 (0.81-1.06) | 0.94 (0.82-1.08) | 0.96 (0.84-1.1) |
| T3 | | 5631.7 | 370 | 6.57 (5.93-7.27) | 0.84 (0.73-0.97) | 0.9 (0.78-1.03) | 0.92 (0.8-1.06) |
| *P* for trend | |  |  |  | 0.015 | 0.119 | 0.250 |
| LF | |  |  |  |  |  |  |
| *P* for interaction | |  |  |  | <0.001 | <0.001 | <0.001 |
| Without dyslipidemia | |  |  |  |  |  |  |
| T1 | | 50595.9 | 1582 | 3.13 (2.98-3.28) | 1 (reference) | 1 (reference) | 1 (reference) |
| T2 | | 50222.8 | 1793 | 3.57 (3.41-3.74) | 0.97 (0.9-1.03) | 0.98 (0.91-1.05) | 0.98 (0.92-1.05) |
| T3 | | 49820.9 | 1863 | 3.74 (3.57-3.91) | 0.89 (0.83-0.95) | 0.91 (0.84-0.97) | 0.92 (0.86-0.98) |
| *P* for trend | |  |  |  | <0.001 | 0.004 | 0.012 |
| With dyslipidemia | |  |  |  |  |  |  |
| T1 | | 5940.9 | 387 | 6.51 (5.9-7.2) | 1 (reference) | 1 (reference) | 1 (reference) |
| T2 | | 5782.4 | 410 | 7.09 (6.44-7.81) | 1.01 (0.88-1.16) | 1 (0.87-1.15) | 1.02 (0.88-1.17) |
| T3 | | 6050.5 | 431 | 7.12 (6.48-7.83) | 0.9 (0.78-1.03) | 0.93 (0.81-1.07) | 0.96 (0.83-1.1) |
| *P* for trend | |  |  |  | 0.127 | 0.289 | 0.544 |
| HF | |  |  |  |  |  |  |
| *P* for interaction | |  |  |  | <0.001 | <0.001 | 0.003 |
| Without dyslipidemia | |  |  |  |  |  |  |
| T1 | | 48758.1 | 1957 | 4.01 (3.84-4.2) | 1 (reference) | 1 (reference) | 1 (reference) |
| T2 | | 50775.2 | 1732 | 3.41 (3.25-3.58) | 0.87 (0.82-0.93) | 0.91 (0.85-0.97) | 0.93 (0.87-0.99) |
| T3 | | 51106.3 | 1549 | 3.03 (2.88-3.19) | 0.81 (0.75-0.86) | 0.86 (0.8-0.92) | 0.9 (0.84-0.96) |
| *P* for trend | |  |  |  | <0.001 | <0.001 | 0.002 |
| With dyslipidemia | |  |  |  |  |  |  |
| T1 | | 6843.8 | 539 | 7.88 (7.24-8.57) | 1 (reference) | 1 (reference) | 1 (reference) |
| T2 | | 5900.5 | 393 | 6.66 (6.03-7.35) | 0.8 (0.7-0.91) | 0.84 (0.73-0.96) | 0.85 (0.75-0.97) |
| T3 | | 5029.5 | 296 | 5.89 (5.25-6.6) | 0.77 (0.67-0.89) | 0.83 (0.72-0.97) | 0.86 (0.74-0.998) |
| *P* for trend | |  |  |  | <0.001 | 0.008 | 0.027 |
| LF norm (n.u.) | |  |  |  |  |  |  |
| *P* for interaction | |  |  |  | <0.001 | 0.012 | 0.105 |
| Without dyslipidemia | |  |  |  |  |  |  |
| T1 | | 51678 | 1359 | 2.63 (2.49-2.77) | 1 (reference) | 1 (reference) | 1 (reference) |
| T2 | | 50435.6 | 1800 | 3.57 (3.41-3.74) | 1.11 (1.03-1.19) | 1.11 (1.03-1.19) | 1.1 (1.02-1.18) |
| T3 | | 48525.9 | 2079 | 4.28 (4.1-4.47) | 1.13 (1.06-1.22) | 1.07 (0.999-1.15) | 1.05 (0.97-1.12) |
| *P* for trend | |  |  |  | 0.001 | 0.092 | 0.342 |
| With dyslipidemia | |  |  |  |  |  |  |
| T1 | | 5109.5 | 278 | 5.44 (4.84-6.12) | 1 (reference) | 1 (reference) | 1 (reference) |
| T2 | | 5673.4 | 395 | 6.96 (6.31-7.68) | 1.07 (0.92-1.25) | 1.04 (0.89-1.22) | 1.03 (0.88-1.21) |
| T3 | | 6990.9 | 555 | 7.94 (7.31-8.63) | 1.14 (0.99-1.33) | 1.1 (0.94-1.27) | 1.08 (0.93-1.26) |
| *P* for trend | |  |  |  | 0.072 | 0.213 | 0.282 |
| HF norm (n.u.) | |  |  |  |  |  |  |
| *P* for interaction | |  |  |  | <0.001 | 0.016 | 0.107 |
| Without dyslipidemia | |  |  |  |  |  |  |
| T1 | | 48412.6 | 2052 | 4.24 (4.06-4.43) | 1 (reference) | 1 (reference) | 1 (reference) |
| T2 | | 50295.7 | 1813 | 3.6 (3.44-3.77) | 0.998 (0.94-1.06) | 1.06 (0.99-1.13) | 1.07 (1.002-1.14) |
| T3 | | 51931.3 | 1373 | 2.64 (2.51-2.79) | 0.9 (0.84-0.96) | 0.95 (0.89-1.02) | 0.98 (0.91-1.05) |
| *P* for trend | |  |  |  | 0.005 | 0.303 | 0.751 |
| With dyslipidemia | |  |  |  |  |  |  |
| T1 | | 7002 | 551 | 7.87 (7.24-8.55) | 1 (reference) | 1 (reference) | 1 (reference) |
| T2 | | 5699.8 | 396 | 6.95 (6.3-7.67) | 0.94 (0.83-1.07) | 0.96 (0.84-1.09) | 0.96 (0.85-1.1) |
| T3 | | 5072 | 281 | 5.54 (4.93-6.23) | 0.89 (0.77-1.03) | 0.93 (0.8-1.08) | 0.94 (0.81-1.09) |
| *P* for trend | |  |  |  | 0.115 | 0.333 | 0.419 |
| LF/HF ratio | |  |  |  |  |  |  |
| *P* for interaction | |  |  |  | <0.001 | 0.031 | 0.256 |
| Without dyslipidemia | |  |  |  |  |  |  |
| T1 | | 48561.3 | 1271 | 2.62 (2.48-2.77) | 1 (reference) | 1 (reference) | 1 (reference) |
| T2 | | 51009.8 | 1780 | 3.49 (3.33-3.66) | 1.09 (1.01-1.17) | 1.09 (1.01-1.17) | 1.07 (0.995-1.15) |
| T3 | | 51068.4 | 2187 | 4.28 (4.11-4.47) | 1.14 (1.06-1.22) | 1.08 (1.005-1.16) | 1.05 (0.98-1.13) |
| *P* for trend | |  |  |  | <0.001 | 0.058 | 0.239 |
| With dyslipidemia | |  |  |  |  |  |  |
| T1 | | 4798.6 | 259 | 5.4 (4.78-6.1) | 1 (reference) | 1 (reference) | 1 (reference) |
| T2 | | 5593.9 | 391 | 6.99 (6.33-7.72) | 1.07 (0.91-1.25) | 1.03 (0.87-1.2) | 1.02 (0.87-1.2) |
| T3 | | 7381.3 | 578 | 7.83 (7.22-8.5) | 1.13 (0.97-1.31) | 1.07 (0.92-1.25) | 1.06 (0.91-1.24) |
| *P* for trend | |  |  |  | 0.115 | 0.326 | 0.417 |

Abbreviations: AST, aspartate transaminase; BMI, body mass index; CI, confidence interval; HF, high-frequency; HOMA-IR, homeostatic model assessment-Insulin resistance; HR, hazard ratio; hs-CRP, high-sensitivity c-reactive protein; LDL-C, low-density lipoprotein cholesterol; LF, low-frequency; RMSSD, root mean square difference; SDNN, standard deviation of the normal-to-normal interval; TP, total power.

^a^ Cox proportional hazards regression models were used to estimate HRs and 95% CIs.

^b^ Model 1 was adjusted for age, sex, body mass index, current smoking, alcohol intake, regular exercise, aspartate transaminase, low-density lipoprotein cholesterol levels, and systolic blood pressure.

^c^ Model 2 is was adjusted for the same parameters as model 1 plus high-sensitivity C-reactive protein, homeostasis model assessment for insulin resistance score, and presence of diabetes.

**Supplementary Table 4** Risk of incident non-alcoholic fatty liver disease according to tertiles of heart rate variability indices.

|  | **Number of subjects** | **Person-years** | **Incident cases** | **Incidence rate**  **(per 100 person-years)** | **Age- and sex-adjusted HR (95% CI)^a^** | **Multivariate-adjusted HR (95% CI)^a^** | |
| --- | --- | --- | --- | --- | --- | --- | --- |
|  |  |  |  |  |  | **Model 1^b^** | **Model 2^c^** |
| Total subjects | 33,899 | 168,413 | 6,466 | 3.84 (3.75-3.93) |  |  |  |
| Heart rate |  |  |  |  |  |  |  |
| T1 | 10,959 | 54,715 | 1,997 | 3.65 (3.49-3.81) | 1 (reference) | 1 (reference) | 1 (reference) |
| T2 | 11,627 | 57,842 | 2,163 | 3.74 (3.59-3.90) | 1.19 (1.12-1.26) | 1.14 (1.08-1.22) | 1.12 (1.05-1.19) |
| T3 | 11,313 | 55,856 | 2,306 | 4.13 (3.96-4.30) | 1.38 (1.30-1.47) | 1.28 (1.21-1.36) | 1.22 (1.15-1.30) |
| *P* for trend |  |  |  |  | <0.001 | <0.001 | <0.001 |
| SDNN (ms) |  |  |  |  |  |  |  |
| T1 | 11,299 | 56,133 | 2,254 | 4.02 (3.85-4.18) | 1 (reference) | 1 (reference) | 1 (reference) |
| T2 | 11,228 | 56,273 | 2,113 | 3.75 (3.60-3.92) | 0.88 (0.83-0.93) | 0.92 (0.86-0.97) | 0.94 (0.88-0.99) |
| T3 | 11,372 | 56,007 | 2,099 | 3.75 (3.59-3.91) | 0.83 (0.78-0.88) | 0.87 (0.82-0.93) | 0.90 (0.85-0.96) |
| *P* for trend |  |  |  |  | <0.001 | <0.001 | 0.001 |
| RMSSD (ms) |  |  |  |  |  |  |  |
| T1 | 11,214 | 55,455 | 2,502 | 4.51 (4.34-4.69) | 1 (reference) | 1 (reference) | 1 (reference) |
| T2 | 11,348 | 56,650 | 2,134 | 3.77 (3.61-3.93) | 0.84 (0.79-0.89) | 0.89 (0.84-0.94) | 0.91 (0.85-0.96) |
| T3 | 11,337 | 56,307 | 1,830 | 3.25 (3.10-3.40) | 0.74 (0.70-0.79) | 0.79 (0.75-0.84) | 0.83 (0.78-0.88) |
| *P* for trend |  |  |  |  | <0.001 | <0.001 | <0.001 |
| TP (ms^2^) |  |  |  |  |  |  |  |
| T1 | 11,294 | 56,068 | 2,243 | 4.00 (3.84-4.17) | 1 (reference) | 1 (reference) | 1 (reference) |
| T2 | 11,304 | 56,514 | 2,121 | 3.75 (3.60-3.92) | 0.89 (0.84-0.95) | 0.92 (0.86-0.97) | 0.93 (0.88-0.99) |
| T3 | 11,301 | 55,831 | 2,102 | 3.76 (3.61-3.93) | 0.84 (0.79-0.89) | 0.89 (0.84-0.95) | 0.91 (0.86-0.97) |
| *P* for trend |  |  |  |  | <0.001 | <0.001 | 0.004 |
| LF (ms^2^) |  |  |  |  |  |  |  |
| T1 | 11,296 | 56537 | 1969 | 3.50 (3.30-3.60) | 1 (reference) | 1 (reference) | 1 (reference) |
| T2 | 11,302 | 56005 | 2203 | 3.90 (3.80-4.10) | 0.97 (0.91-1.03) | 0.98 (0.93-1.05) | 0.99 (0.94-1.06) |
| T3 | 11,301 | 55871 | 2294 | 4.10 (3.90-4.30) | 0.89 (0.84-0.95) | 0.91 (0.86-0.97) | 0.93 (0.87-0.99) |
| *P* for trend |  |  |  |  | <0.001 | 0.003 | 0.019 |
| HF (ms^2^) |  |  |  |  |  |  |  |
| T1 | 11,296 | 55602 | 2496 | 4.50 (4.30-4.70) | 1 (reference) | 1 (reference) | 1 (reference) |
| T2 | 11,302 | 56676 | 2125 | 3.80 (3.60-3.90) | 0.85 (0.81-0.91) | 0.90 (0.85-0.95) | 0.92 (0.86-0.97) |
| T3 | 11,301 | 56136 | 1845 | 3.30 (3.10-3.40) | 0.80 (0.75-0.85) | 0.86 (0.81-0.91) | 0.89 (0.83-0.94) |
| *P* for trend |  |  |  |  | <0.001 | <0.001 | <0.001 |
| LF norm |  |  |  |  |  |  |  |
| T1 | 11,280 | 56,788 | 1,637 | 2.88 (2.75-3.03) | 1 (reference) | 1 (reference) | 1 (reference) |
| T2 | 11,313 | 56,109 | 2,195 | 3.91 (3.75-4.08) | 1.11 (1.04-1.18) | 1.09 (1.03-1.17) | 1.09 (1.02-1.16) |
| T3 | 11,306 | 55,517 | 2,634 | 4.74 (4.57-4.93) | 1.15 (1.08-1.22) | 1.08 (1.01-1.15) | 1.06 (0.99-1.13) |
| *P* for trend |  |  |  |  | <0.001 | 0.038 | 0.158 |
| HF norm |  |  |  |  |  |  |  |
| T1 | 11,278 | 55,415 | 2,603 | 4.70 (4.52-4.88) | 1 (reference) | 1 (reference) | 1 (reference) |
| T2 | 11,292 | 55,995 | 2,209 | 3.94 (3.78-4.11) | 0.98 (0.93-1.04) | 1.03 (0.98-1.10) | 1.05 (0.99-1.11) |
| T3 | 11,329 | 57,003 | 1,654 | 2.90 (2.77-3.04) | 0.89 (0.83-0.95) | 0.95 (0.89-1.01) | 0.97 (0.91-1.03) |
| *P* for trend |  |  |  |  | <0.001 | 0.169 | 0.465 |
| LF/HF ratio |  |  |  |  |  |  |  |
| T1 | 10,601 | 53,360 | 1,530 | 2.87 (2.73-3.01) | 1 (reference) | 1 (reference) | 1 (reference) |
| T2 | 11,389 | 56,604 | 2,171 | 3.84 (3.68-4.00) | 1.09 (1.02-1.16) | 1.07 (1.00-1.15) | 1.06 (0.99-1.14) |
| T3 | 11,909 | 58,450 | 2,765 | 4.73 (4.56-4.91) | 1.14 (1.07-1.22) | 1.08 (1.01-1.15) | 1.06 (0.99-1.13) |
| *P* for trend |  |  |  |  | <0.001 | 0.033 | 0.142 |

Abbreviations: CI, confidence interval; HF, high-frequency; HF norm, normalized high-frequency; HR, hazard ratio; LF, low-frequency; LF norm, normalized low-frequency; RMSSD, root mean square difference; SDNN, standard deviation of the normal-to-normal interval; TP, total power.

^a^ Cox proportional hazards regression models were used to estimate HRs and 95% CIs.

^b^ Model 1 was adjusted for age, sex, body mass index, current smoking, alcohol intake, regular exercise, aspartate transaminase, low-density lipoprotein cholesterol levels, and systolic blood pressure.

^c^ Model 2 was adjusted for the same parameters as model 1 plus high-sensitivity C-reactive protein, homeostasis model assessment for insulin resistance score, and presence of diabetes.

**Supplementary Table 5** Risk of incident non-alcoholic fatty liver disease according to change of HRV measurement in subjects who underwent follow-up HRV exam within two years.

|  |  | **V1 exam** | **V2 exam** | **No. of**  **subjects** | **Age- and sex-adjusted**  **HR (95% CI)^a^** | **Multivariate-adjusted HR (95% CI)^a^** | |
| --- | --- | --- | --- | --- | --- | --- | --- |
|  |  |  |  |  |  | **Model 1^b^** | **Model 2^c^** |
| Heart rate | Group 0 | V1 <64 | V2 <64 | 4,817 | 1 (reference) | 1 (reference) | 1 (reference) |
|  | Group 1 | V1 <64 | V2 ≥64 | 1,792 | 1.48 (1.2-1.81) | 1.49 (1.21-1.83) | 1.45 (1.18-1.77) |
|  | Group 2 | V1 ≥64 | V2 <64 | 1,809 | 1.06 (0.84-1.34) | 0.96 (0.76-1.22) | 0.92 (0.73-1.17) |
|  | Group 3 | V1 ≥64 | V2 ≥64 | 5,525 | 1.54 (1.32-1.80) | 1.45 (1.24-1.70) | 1.37 (1.17-1.60) |
| SDNN (ms) | Group 0 | V1 <41.7 | V2 <40.9 | 3,452 | 1 (reference) | 1 (reference) | 1 (reference) |
|  | Group 1 | V1 <41.7 | V2 ≥40.9 | 1,580 | 0.92 (0.72-1.16) | 0.96 (0.75-1.21) | 0.99 (0.78-1.26) |
|  | Group 2 | V1 ≥41.7 | V2 <40.9 | 1,381 | 0.95 (0.74-1.21) | 0.99 (0.78-1.27) | 1.07 (0.84-1.37) |
|  | Group 3 | V1 ≥41.7 | V2 ≥40.9 | 3,260 | 0.76 (0.62-0.93) | 0.85 (0.69-1.04) | 0.92 (0.75-1.12) |
| RMSSD (ms) | Group 0 | V1 <37.1 | V2 <35.8 | 3,693 | 1 (reference) | 1 (reference) | 1 (reference) |
|  | Group 1 | V1 <37.1 | V2 ≥35.8 | 1,367 | 0.65 (0.49-0.85) | 0.72 (0.54-0.95) | 0.77 (0.58-1.03) |
|  | Group 2 | V1 ≥37.1 | V2 <35.8 | 1,138 | 1.10 (0.87-1.39) | 1.14 (0.90-1.45) | 1.24 (0.98-1.57) |
|  | Group 3 | V1 ≥37.1 | V2 ≥35.8 | 3,475 | 0.67 (0.55-0.82) | 0.76 (0.62-0.93) | 0.86 (0.70-1.05) |
| TP (ms^2^) | Group 0 | V1 <1,149.1 | V2 <1,069.2 | 3,265 | 1 (reference) | 1 (reference) | 1 (reference) |
|  | Group 1 | V1 <1,149.1 | V2 ≥1,069.2 | 1,714 | 0.84 (0.65-1.07) | 0.98 (0.76-1.26) | 1.02 (0.80-1.32) |
|  | Group 2 | V1 ≥1,149.1 | V2 <1,069.2 | 1,570 | 0.96 (0.76-1.21) | 1.07 (0.84-1.35) | 1.11 (0.88-1.41) |
|  | Group 3 | V1 ≥1,149.1 | V2 ≥1,069.2 | 3,124 | 0.91 (0.75-1.12) | 1.02 (0.83-1.25) | 1.08 (0.88-1.32) |
| LF | Group 0 | V1 <248.7 | V2 <230.8 | 3,177 | 1 (reference) | 1 (reference) | 1 (reference) |
|  | Group 1 | V1 <248.7 | V2 ≥230.8 | 1,782 | 0.98 (0.77-1.26) | 1.07 (0.83-1.37) | 1.06 (0.83-1.36) |
|  | Group 2 | V1 ≥248.7 | V2 <230.8 | 1,659 | 1.03 (0.80-1.31) | 1.13 (0.88-1.44) | 1.12 (0.88-1.43) |
|  | Group 3 | V1 ≥248.7 | V2 ≥230.8 | 3,055 | 1.01 (0.82-1.24) | 1.08 (0.88-1.33) | 1.08 (0.88-1.33) |
| HF | Group 0 | V1 <342.3 | V2 <303.7 | 3,606 | 1 (reference) | 1 (reference) | 1 (reference) |
|  | Group 1 | V1 <342.3 | V2 ≥303.7 | 1,422 | 0.69 (0.52-0.9) | 0.82 (0.63-1.08) | 0.88 (0.67-1.15) |
|  | Group 2 | V1 ≥342.3 | V2 <303.7 | 1,228 | 1.06 (0.84-1.34) | 1.14 (0.90-1.44) | 1.17 (0.93-1.49) |
|  | Group 3 | V1 ≥342.3 | V2 ≥303.7 | 3,417 | 0.74 (0.61-0.9) | 0.84 (0.69-1.03) | 0.90 (0.74-1.10) |
| LF norm (n.u.) | Group 0 | V1 <42.4 | V2 <43.1 | 3,069 | 1 (reference) | 1 (reference) | 1 (reference) |
|  | Group 1 | V1 <42.4 | V2 ≥43.1 | 1,719 | 1.59 (1.24-2.06) | 1.51 (1.17-1.95) | 1.52 (1.17-1.96) |
|  | Group 2 | V1 ≥42.4 | V2 <43.1 | 1,763 | 1.02 (0.76-1.36) | 0.99 (0.75-1.33) | 1.01 (0.75-1.34) |
|  | Group 3 | V1 ≥42.4 | V2 ≥43.1 | 3,122 | 1.52 (1.21-1.90) | 1.39 (1.10-1.74) | 1.29 (1.02-1.62) |
| HF norm (n.u.) | Group 0 | V1 <57.2 | V2 <56.6 | 3,100 | 1 (reference) | 1 (reference) | 1 (reference) |
|  | Group 1 | V1 <57.2 | V2 ≥56.6 | 1,753 | 0.72 (0.56-0.92) | 0.77 (0.60-0.99) | 0.83 (0.65-1.06) |
|  | Group 2 | V1 ≥57.2 | V2 <56.6 | 1,734 | 1.04 (0.84-1.29) | 1.09 (0.88-1.35) | 1.18 (0.95-1.47) |
|  | Group 3 | V1 ≥57.2 | V2 ≥56.6 | 3,086 | 0.67 (0.54-0.84) | 0.74 (0.59-0.93) | 0.79 (0.63-0.99) |
| LF/HF ratio | Group 0 | V1 <0.7 | V2 <0.8 | 2,804 | 1 (reference) | 1 (reference) | 1 (reference) |
|  | Group 1 | V1 <0.7 | V2 ≥0.8 | 1,486 | 1.61 (1.22-2.14) | 1.53 (1.16-2.03) | 1.52 (1.14-2.01) |
|  | Group 2 | V1 ≥0.7 | V2 <0.8 | 1,971 | 1.06 (0.79-1.41) | 1.02 (0.76-1.36) | 1.02 (0.77-1.37) |
|  | Group 3 | V1 ≥0.7 | V2 ≥0.8 | 3,412 | 1.60 (1.26-2.02) | 1.45 (1.14-1.85) | 1.37 (1.07-1.74) |

Abbreviations: CI, confidence interval; HF, high-frequency; HF norm, normalized high-frequency; HR, hazard ratio; LF, low-frequency; LF norm, normalized low-frequency; RMSSD, root mean square difference; SDNN, standard deviation of the normal-to-normal interval; TP, total power.

^a^ Cox proportional hazards regression models were used to estimate HRs and 95% CIs.

^b^ Model 1 was adjusted for age, sex, body mass index, current smoking, alcohol intake, regular exercise, aspartate transaminase, low-density lipoprotein cholesterol levels, and systolic blood pressure.

^c^ Model 2 was adjusted for the same parameters as model 1 plus high-sensitivity C-reactive protein, homeostasis model assessment for insulin resistance score, and presence of diabetes.
